# Improving environmental cleaning quality and consistency in a multi-campus hospital using a computer vision-assisted mobile application: a prospective interventional study

**DOI:** 10.1186/s13756-026-01803-x

**Published:** 2026-08-10

**Authors:** Pingping Wang, Jin Luo, Lan Chen, Zheng Xiang, Yu Wang, Yujun He, Qun Sun, Jing Chen, Qian Xiang

**Affiliations:** 1 Healthcare-Associated Infection Control Center, School of Medicine, Sichuan Academy of Medical Sciences, Sichuan Provincial People’s Hospital, University of Electronic Science and Technology of China, Chengdu, China; 2 Logistics Support Department, School of Medicine, Sichuan Academy of Medical Sciences, Sichuan Provincial People’s Hospital, University of Electronic Science and Technology of China, Chengdu, China; 3 Planning, Finance, and Quality Assurance Department, Irradiation Preservation Key Laboratory of Sichuan Province, Chengdu Institute of Food Inspection, Chengdu, Sichuan China; 4 Human Resources Department, 903 Hospital, Jiangyou, Mianyang, Sichuan China; 5 Department of Healthcare-Associated Infection Management, 903 Hospital, Jiangyou, Mianyang, Sichuan China; 6 Business Management and Information Technology Department, Irradiation Preservation Key Laboratory of Sichuan Province, Chengdu Institute of Food Inspection, Chengdu, Sichuan China

**Keywords:** Computer vision, Cleaning quality, Environmental hygiene, Healthcare-associated infection, Multi-campus hospital, PDCA, Quality improvement

## Abstract

**Background:**

Consistent environmental cleaning is essential for preventing healthcare-associated infections, yet achieving uniform standards across multi-campus hospital systems is difficult. Conventional methods, such as manual visual inspection and traditional fluorescent marking, are inherently subjective, qualitative, and prone to observer bias. This study assessed whether a computer vision-assisted mobile application embedded in a technology-empowered Plan-Do-Check-Act (T-PDCA) quality improvement model improves cleaning performance and promotes homogeneity across campuses.

**Methods:**

We conducted a 17-week prospective interventional study (June–October 2025) in a tertiary hospital system with four geographically dispersed campuses in Southwest China. A smartphone application generated an algorithm-derived fluorescent marker removal rate (FMRR) score by analyzing ultraviolet fluorescence images captured before and after routine cleaning and provided real-time quantitative feedback. The intervention was implemented within a T-PDCA framework, including standardized training, blinded fluorescent marking, algorithm-based performance assessment, and data-driven feedback. Cleaning performance was graded using predefined FMRR thresholds. Temporal trends, between-campus homogeneity, and differences by unit type and surface material were evaluated using nonparametric statistics.

**Results:**

A total of 2,457 valid fluorescent marker samples were analyzed during the study period. The overall weekly mean FMRR was 83.91% in Week 1 and remained above 90% from approximately Week 4 onward, with a significant upward trend across the study period (Spearman’s *ρ* = 0.615, *P* = 0.01). Performance variability decreased substantially, with the weekly standard deviation declining from 29.72 to 9.59%, indicating improved homogeneity. Campuses with lower baseline performance showed improvement, while high-performing campuses maintained relatively stable cleaning quality. Significant differences in FMRR were observed across hospital units and surface materials; notably, intensive care units, outpatient departments, and surfaces made of matte plastic or wood demonstrated lower and more variable performance.

**Conclusions:**

Integrating a computer vision-assisted mobile monitoring system into a T-PDCA quality improvement framework was associated with improved environmental cleaning performance and enhanced homogeneity across a multi-campus hospital system. This approach may support standardized, real-time monitoring of targeted high-touch surfaces and provide a practical tool for improving environmental cleaning management in multi-campus hospital systems.

**Supplementary Information:**

The online version contains supplementary material available at https://doi.org/10.1186/s13756-026-01803-x.

## Introduction

Environmental cleaning and disinfection are fundamental measures for interrupting pathogen transmission and preventing healthcare-associated infections [1, 2]. To address growing healthcare demands and enhance regional capacity, large public hospitals in China are increasingly adopting the multi-campus hospital system model [3]. However, this expansion introduces significant management challenges, including geographical dispersion, reliance on diverse outsourcing teams, and variability in staff competency, which collectively hinder the consistent implementation of cleaning standards [4]. Consequently, achieving consistency in environmental hygiene quality across campuses has become a critical goal for infection prevention and control (IPC), with direct implications for patient safety.

Addressing these challenges and ensuring consistent hygiene standards hinges upon the ability to accurately measure and monitor cleaning performance. However, effective management remains constrained by limitations in current monitoring methodologies. At present, the assessment of environmental cleaning relies primarily on manual visual inspection or traditional fluorescent marking methods [5]. Manual visual inspection is inherently subjective and lacks standardized quantitative criteria, rendering it vulnerable to variations in individual experience, attention levels, visual fatigue, and ambient lighting conditions [6]. Similarly, while traditional fluorescent marking is more objective than visual inspection, it remains largely qualitative, sensitive to lighting variations, and dependent on the subjective interpretation of results [7]. These limitations impede the generation of the accurate, timely, and actionable data necessary to support systematic improvements in environmental hygiene.

In recent years, the convergence of mobile health technologies and computer vision algorithms has created new opportunities to overcome these obstacles. The integration of computer vision techniques with smartphone-based platforms enables the transformation of unstructured image data into objective, quantifiable metrics, thereby facilitating standardized and reproducible assessment [8]. By reducing reliance on human judgment, such approaches have the potential to minimize observer bias while enabling real-time data capture, transmission, and analysis via mobile networks. Although computer vision-assisted applications have been increasingly adopted in clinical diagnostic settings [9, 10], their application in environmental hygiene monitoring and IPC remains limited and under-studied.

Despite their technical potential, computer vision-assisted tools alone are insufficient to drive sustained improvement unless systematically embedded within established quality improvement frameworks that translate data into continuous action. Continuous quality improvement frameworks, such as the Plan-Do-Check-Act (PDCA) cycle, are widely applied in hospital IPC [11, 12]. However, the efficacy of the PDCA cycle fundamentally depends on the availability of objective and timely data during the Check phase to inform evidence-based decisions in the subsequent Act phase. To operationalize the integration of digital monitoring and quality management, we proposed and implemented a technology-empowered PDCA (T-PDCA) model that incorporates a computer vision-assisted mobile application into the quality improvement cycle. By reducing reliance on experience-based judgment and applying a standardized algorithm-assisted scoring procedure, this approach enables more consistent performance assessment and continuous feedback. In this 17-week prospective interventional study, we evaluated whether this computer vision-assisted mobile application could improve overall environmental cleaning performance and promote the homogeneity of environmental cleaning quality across a multi-campus hospital system.

## Methods

### Study design and setting

We conducted a prospective interventional study over a 17-week period from June to October 2025 in a large tertiary hospital in Southwest China. The hospital operates under a group-based management model comprising four geographically dispersed campuses (Campuses A, B, C, and D), with a total capacity of approximately 4,500 inpatient beds. The intervention targeted key clinical and public areas across all campuses, including general wards, intensive care units (ICUs), operating rooms, outpatient departments, treatment rooms, and public restrooms. The study design and reporting adhered to the Standards for Quality Improvement Reporting Excellence (SQUIRE 2.0) guidelines [13].

### Intervention tool

The intervention used a custom-developed, computer vision-assisted monitoring system deployed as a WeChat mini-program (Tencent, Shenzhen, China). The system consisted of three core functional modules:

1. Image acquisition module: This module captured fluorescent marker images using a smartphone camera under excitation from an ultraviolet-A (UVA) light-emitting diode (LED) source with a wavelength of 390–400 nm. Images were acquired using smartphones from three brands, including the iPhone 14 Pro, iPhone 15 Pro, and iPhone 16 Pro (Apple Inc., Cupertino, CA, USA); Huawei Mate 50 Pro, Mate 60 Pro, Mate 70 Pro, P60, and Nova 12 (Huawei Technologies, Shenzhen, China); and HONOR 80 (Honor Device Co., Shenzhen, China). Most devices were flagship-tier smartphones; however, differences in camera sensors and image-processing pipelines across models could not be completely excluded. To reduce device-related variability, image acquisition procedures were standardized across all devices. Automatic image-enhancement functions, including automatic high dynamic range, night mode, computational sharpening, and noise reduction, were disabled when technically available. Imaging parameters, including exposure, focus, and ISO sensitivity, were manually locked where technically available. Images were stored in standard Joint Photographic Experts Group (JPEG) format before analysis. Image acquisition was performed inside a customized light-shielding enclosure under a standardized 390–400 nm UVA-LED excitation source. The algorithm additionally performed background-based intensity normalization before segmentation to reduce residual differences in image brightness and sensor response between devices. Because formal validation across a wider range of smartphone models was not performed, the reported results should be interpreted as performance under standardized acquisition conditions using the specified devices rather than as evidence of device-independent validity.
2. Algorithm processing module: This module incorporated a specialized rule-based computer vision algorithm optimized for the 390–400 nm spectral band. Rather than employing computationally intensive deep learning models, the system utilized a robust adaptive thresholding segmentation approach to automatically identify fluorescent pixel regions. This design supported efficient and reproducible processing on the specified mobile devices used in this study. By comparing the fluorescent pixel area before cleaning (*A*_*pre*_) and after cleaning (*A*_*post*_), the system automatically calculated the fluorescent marker removal rate (FMRR), defined as:
  $$ {\mathrm{FMRR}} = \left( {1 - \frac{{A_{{{\mathrm{post}}}} }}{{A_{{{\mathrm{pre}}}} }}} \right) \times 100\% $$

This algorithm-assisted approach reduced the subjectivity associated with visual estimation and enabled standardized calculation of an algorithm-derived semi-quantitative FMRR score ranging from 0 to 100%.

The algorithm employed a tiered evaluation logic to improve reproducibility and inter-operator consistency. As a pre-processing step, background non-fluorescent regions were sampled to establish a baseline luminance reference, and each image was mapped onto a standardized grayscale space. Following adaptive thresholding, morphological opening and closing operations were applied to reduce isolated noise pixels and JPEG compression artifacts. Three evaluation regimes were then defined. First, when no residual fluorescence above the segmentation threshold was detected, the surface was assigned an FMRR of 100%. Second, when residual fluorescence was clearly present and quantifiable, the FMRR was calculated as (1 − residual fluorescent area / initial marker area) × 100%. Third, when residual fluorescence was present but its area could not be reliably segmented because of specular reflection, blurred boundaries, or thresholding ambiguity, the system assigned a predefined partial-removal score of 60%. This rule was specified before analysis and was intended to avoid overestimating near-complete but uncertain removal. Therefore, the concentration of observations around 60% reflects an algorithm-defined partial-removal tier rather than a natural continuous distribution. The FMRR should therefore be interpreted as an algorithm-derived cleaning performance score rather than a purely continuous pixel-area measurement in all cases. The distribution of samples across these tiers is summarized in Additional file 1.

3. Cloud synchronization module: The cloud synchronization module was designed to enable real-time transmission of monitoring data to a central server. The collected data consisted of FMRR values, timestamps, geolocation information, surface material types, and on-site images. This approach improved data integrity, traceability, and consistency across monitoring sites.

**Fig. 1:**
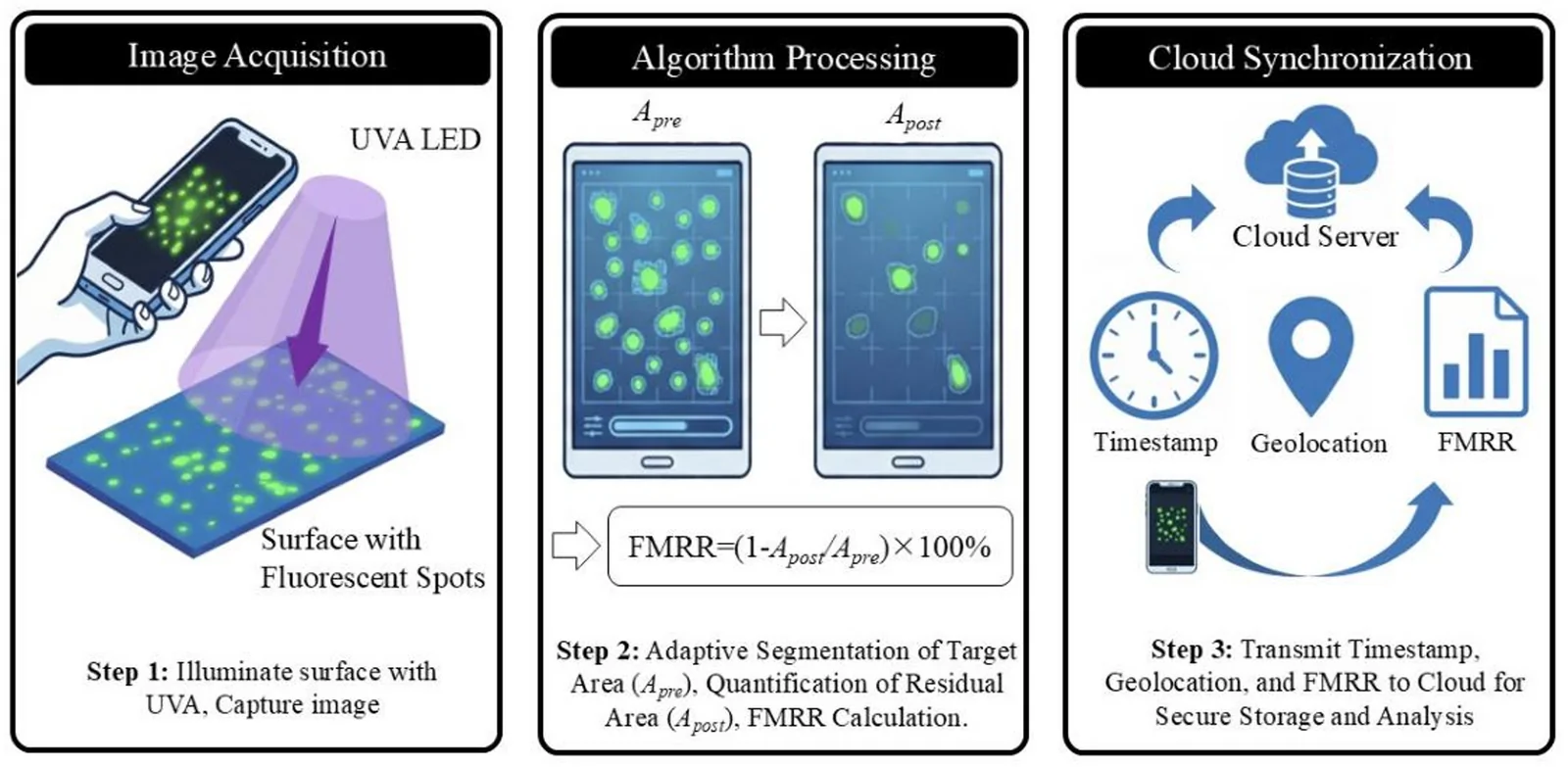
Schematic workflow of the smartphone-based computer vision monitoring system. This figure illustrates the stepwise process of the monitoring system using a smartphone to track and analyze a surface with fluorescent spots. Step 1 (Image Acquisition): The surface is illuminated using a UVA-LED, and the image is captured using a smartphone. Step 2 (Algorithm Processing): The pre-cleaning fluorescent area (*A*_*pre*_) is segmented, and the post-cleaning residual fluorescent area (*A*_*post*_) is quantified to calculate the FMRR. Step 3 (Cloud Synchronization): The timestamp, geolocation, and FMRR data are transmitted to the cloud server for secure storage and analysis. UVA: ultraviolet-A, LED: light-emitting diode, FMRR: fluorescent marker removal rate

**Fig. 2:**
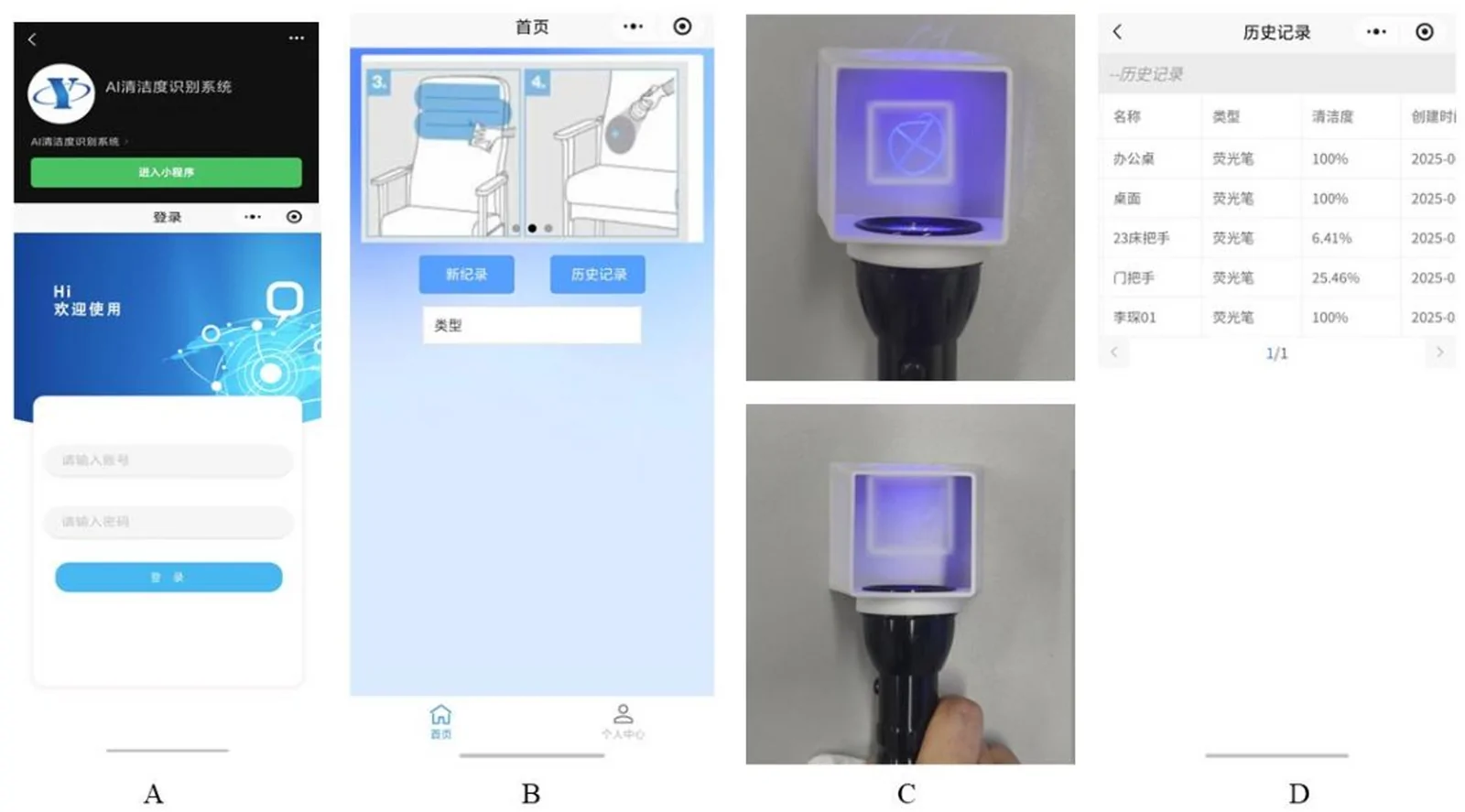
User interface and operational setup of the smartphone-based monitoring system. **A** Login interface of the WeChat mini-program. **B** Home screen featuring visual cleaning guidelines and options for new data entry. **C** The hardware assembly in operation, demonstrating real-time image acquisition under UVA-LED illumination. **D** Historical records interface displaying timestamps, location tags, and calculated FMRR scores for continuous tracking. UVA: ultraviolet-A, LED: light-emitting diode, FMRR: fluorescent marker removal rate

The technical principles of the monitoring system and the application interface are illustrated in Figs. 1 and 2.

The computer vision-assisted monitoring system used in this study was independently developed by our research team. The core system architecture and image recognition methodology are protected by a Chinese invention patent, while the fluorescent image acquisition hardware incorporates a registered utility model patent. In addition, the complete software platform has been officially registered with a computer software copyright. Supporting documentation for these registrations is provided in Additional file 2.

### Intervention implementation

We developed and implemented a T-PDCA model, with the following steps corresponding to the PDCA cycle:

#### Plan (preparation and standardization)

A multidisciplinary project management team was established, comprising members from the hospital infection control, nursing, and logistics departments. Additionally, 23 supervisory staff members from third-party cleaning service providers were recruited as quality control inspectors. Unified cross-campus standard operating procedures were developed, focusing on the standardization of environmental cleaning and disinfection sites, cleaning frequencies, and the operational use of the mobile application. Two rounds of structured training were conducted to ensure that all inspectors were proficient in using the digital monitoring tool.

#### Do (blinded marking and routine cleaning)

Quality control inspectors performed fluorescent marker placement using a single-blind approach. A predefined, campus-specific register of standardized high-touch surface items, including bed rails, call buttons, intravenous poles, door handles, and light switches, was compiled before the study. Each week, inspectors selected a subset of items from this fixed register for marking. No formal a priori sample size calculation was performed because this was an operational quality-improvement study. The number of samples collected at each campus was determined by the number of monitored units and high-touch surface items, campus workload, inspector availability, and workflow feasibility. This selection process was operational rather than computer-randomized. The use of a fixed register reduced on-site discretionary selection and improved reproducibility, although residual selection bias could not be completely excluded because the sampling process was implemented by inspectors. Invisible fluorescent markers (approximately 1–2 cm in diameter) were applied using a fluorescent marking pen. Marker placement was performed either on the day before the scheduled cleaning assessment or on the same day, depending on the workflow of the participating unit. Pre-cleaning ultraviolet verification was not performed to confirm marker integrity, because such verification could have alerted cleaning staff to the presence or location of markers and introduced a Hawthorne effect. Therefore, incidental attenuation of some markers due to routine contact before cleaning could not be completely excluded. Because the markers were invisible under normal lighting and the selected items varied over time, the likelihood that cleaning staff could identify or predict marked locations was considered low. Cleaning staff conducted routine cleaning and disinfection procedures without prior knowledge of the specific marker locations.

#### Check (technology-empowered objective monitoring)

Following cleaning procedures, inspectors used the computer vision-assisted smartphone application to capture images of the marked areas. The application automatically calculated and provided real-time feedback on the FMRR. This algorithm-assisted assessment enabled standardized, machine-driven evaluation, minimizing inter-observer variability in performance scoring.

#### Act (data-driven feedback and continuous improvement)

The system automatically generated weekly visualized monitoring reports, which were distributed to the management teams of the cleaning service providers via workplace communication platforms. For units with FMRR values below the predefined target threshold of 80%, responsible supervisors were required to conduct a root cause analysis and implement corrective actions within 24 h. Recurrent issues, such as cleaning challenges associated with specific surface materials, were addressed through targeted retraining or iterative optimization of the mobile application and workflows, thereby establishing a rapid and continuous quality improvement loop.

### Grading criteria

To facilitate quality management and feedback, cleaning performance was classified into three predefined tiers based on the algorithm-derived FMRR score: Excellent, FMRR ≥ 90%; Qualified, 80% ≤ FMRR < 90%; and Inadequate, FMRR < 80%. An FMRR below 80% triggered corrective action.

These thresholds were used as operational quality-improvement thresholds rather than externally mandated microbiological standards. The 80% cutoff was selected as a pragmatic corrective-action threshold because guidance from the Centers for Disease Control and Prevention for environmental cleaning evaluation uses a thoroughness-of-cleaning rate above 80% as a programmatic benchmark for adjusting the intensity of ongoing monitoring [14]. In addition, a recent study evaluating the cleaning of shared medical equipment defined effective cleaning as removal of at least 80% of ultraviolet fluorescent dots [15]. Therefore, the FMRR < 80% category was considered appropriate for identifying surfaces or units requiring immediate feedback and corrective action. The FMRR ≥ 90% category was selected as a high-reliability performance target rather than as a validated threshold for microbial decontamination or healthcare-associated infection risk reduction.

### Data collection and statistical analysis

All data were cleaned and analyzed using R software (version 4.2.1). Given the non-normal distribution and pronounced ceiling effect observed in the raw FMRR data, descriptive statistics for the overall sample and subgroups are reported as medians and interquartile ranges (IQR).

To capture system-level process changes over time, data were aggregated into weekly means for trend visualization. Because individual-level FMRR values showed a non-normal distribution, a pronounced ceiling effect, and an algorithm-defined 60% partial-removal tier, weekly means were interpreted as process-monitoring indicators rather than as evidence that the individual-level data were normally distributed. For comparisons across groups (e.g., different surface materials or hospital units), the Kruskal–Wallis H test was employed. Where significant overall differences were identified, post hoc pairwise comparisons were conducted using Dunn’s test, with *P* values adjusted using the Bonferroni method. For data visualization, linear fitted plots with 95% confidence intervals (CIs) were used to display temporal trends, and locally estimated scatterplot smoothing (LOESS) was applied to capture nonlinear fluctuations. In campus-stratified analyses, linear regression models were used to evaluate the direction and stability of performance changes. All tests were two-sided, and *P* < 0.05 was considered statistically significant.

To assess the potential influence of unequal sample distribution across campuses, a sensitivity analysis was performed by recomputing the main outcomes after excluding the smallest campus (Campus D). In addition, because a subset of observations was assigned to a predefined 60% partial-removal tier, the overall distribution of algorithm-defined scoring tiers was summarized descriptively.

## Results

### General characteristics

During the 17-week study period, a total of 2,457 valid fluorescent marker samples from environmental surfaces were collected and analyzed. Overall FMRR scores showed a polarized distribution, partly reflecting complete marker removal and the predefined algorithmic scoring tiers. The vast majority of samples (79.89%, 1963/2457) achieved an Excellent rating, with a median FMRR of 100.00% and an IQR of zero. Conversely, 19.13% (470/2457) were rated as Inadequate, while only 0.98% (24/2457) were rated as Qualified. Cleaning performance differed significantly across campuses (*P* < 0.001). Campus B demonstrated the highest observed performance, with the highest Excellent rate (88.40%, 358/405) and the lowest Inadequate rate (11.36%, 46/405). In contrast, Campuses C and D exhibited higher Inadequate rates of 22.56% and 25.86%, respectively; however, these campus-specific estimates should be interpreted cautiously because the sample sizes for Campuses C and D were relatively small (Table 1).

**Table 1:** Distribution of FMRR grades and comparison of cleaning performance levels across four hospital campuses

Campuses	No. of samples	Median FMRR (IQR)^a^	Excellent (≥ 90%)	Qualified (80 to < 90%)	Inadequate (< 80%)	*P* value^b^
A	1830	100.00 (100.00–100.00)	1435 (78.42)	23 (1.26)	372 (20.33)	–
B	405	100.00 (100.00–100.00)	358 (88.40)	1 (0.25)	46 (11.36)	
C	164	100.00 (100.00–100.00)	127 (77.44)	0 (0.00)	37 (22.56)	
D	58	100.00 (84.50–100.00)	43 (74.14)	0 (0.00)	15 (25.86)	
Overall	2457	100.00 (100.00–100.00)	1963 (79.89)	24 (0.98)	470 (19.13)	< 0.001

Values are presented as n (%) unless otherwise indicated. ^a^ IQR: Interquartile Range (P25–P75). ^b^ Calculated using the Kruskal–Wallis H test, indicating significant differences in cleaning performance across campuses. FMRR: fluorescent marker removal rate

### Temporal trends and homogeneity improvement

**Fig. 3:**
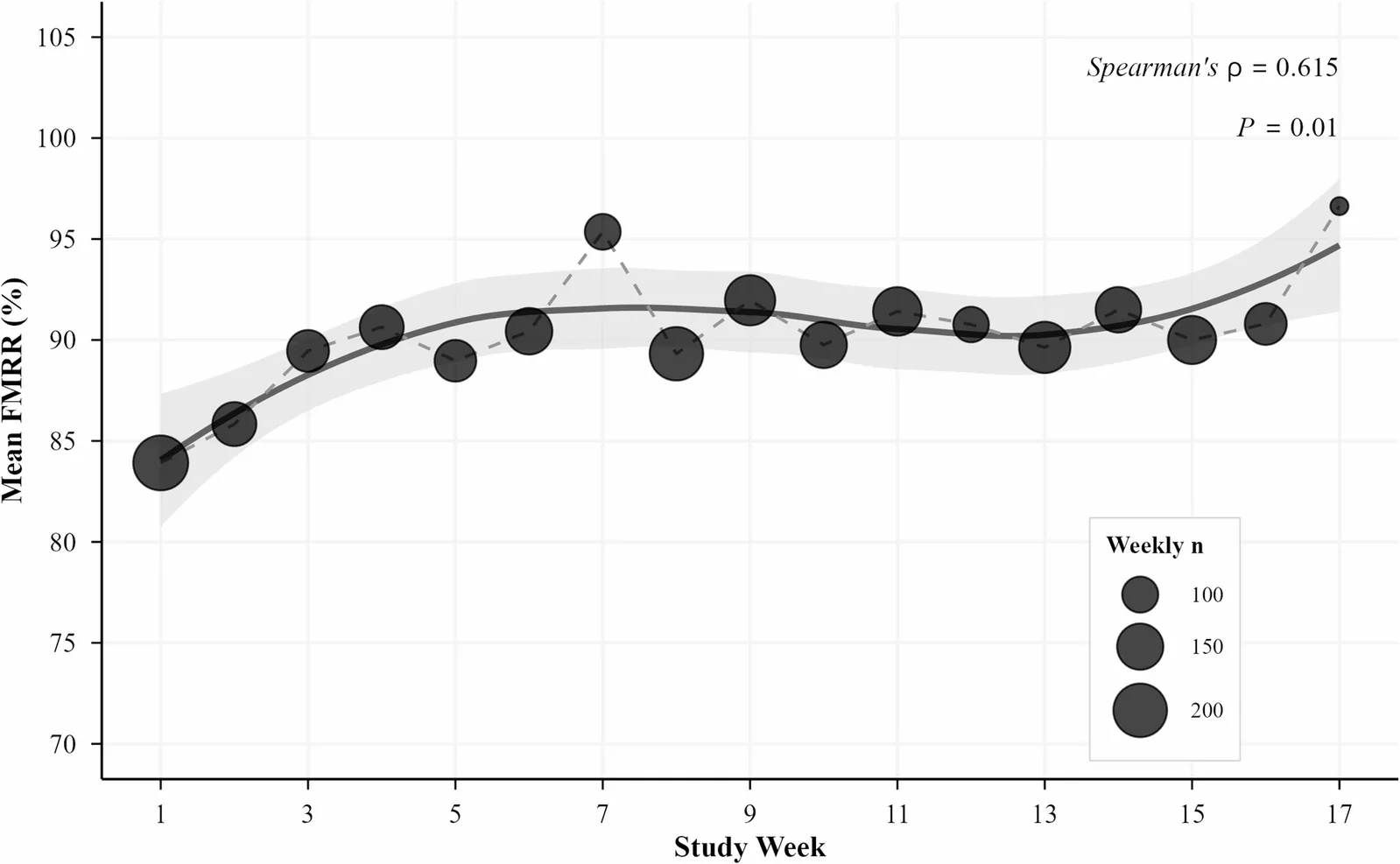
Weekly trend in FMRR and sample distribution during the 17-week intervention period. Each point represents the weekly mean FMRR, and the point size reflects the number of valid samples collected in that week. The gray shaded area indicates the 95% CI around the LOESS fitted curve. Improvement in homogeneity was assessed separately using the decline in weekly SD, rather than the width of the confidence band. Weekly sample sizes were: Week 1, *n* = 210; Week 2, *n* = 137; Week 3, *n* = 126; Week 4, *n* = 183; Week 5, *n* = 148; Week 6, *n* = 191; Week 7, *n* = 149; Week 8, *n* = 156; Week 9, *n* = 117; Week 10, *n* = 158; Week 11, *n* = 129; Week 12, *n* = 126; Week 13, *n* = 124; Week 14, *n* = 167; Week 15, *n* = 124; Week 16, *n* = 147; and Week 17, *n* = 65. FMRR: fluorescent marker removal rate. CI: confidence interval, LOESS: locally estimated scatterplot smoothing, SD: standard deviation

The T-PDCA cycle intervention was associated with improved cleaning performance over time, although the temporal pattern was not strictly linear. As shown in Fig. 3, the weekly mean FMRR was 83.91% in Week 1 and remained slightly above 90% from approximately Week 4 onward. The final-week mean reached 96.63%; however, this value should be interpreted cautiously because Week 17 had the smallest weekly sample size (*n* = 65), partly due to the staggered conclusion of data collection at smaller campuses. Overall, the temporal trend remained statistically significant (Spearman’s *ρ* = 0.615, *P* = 0.01). The standard deviation (SD) of the weekly FMRR decreased from 29.72% initially to 9.59% by the end of the study, suggesting reduced variability in cleaning performance over time. The weekly sample counts across all 17 weeks are reported in the Fig. 3 caption.

**Fig. 4:**
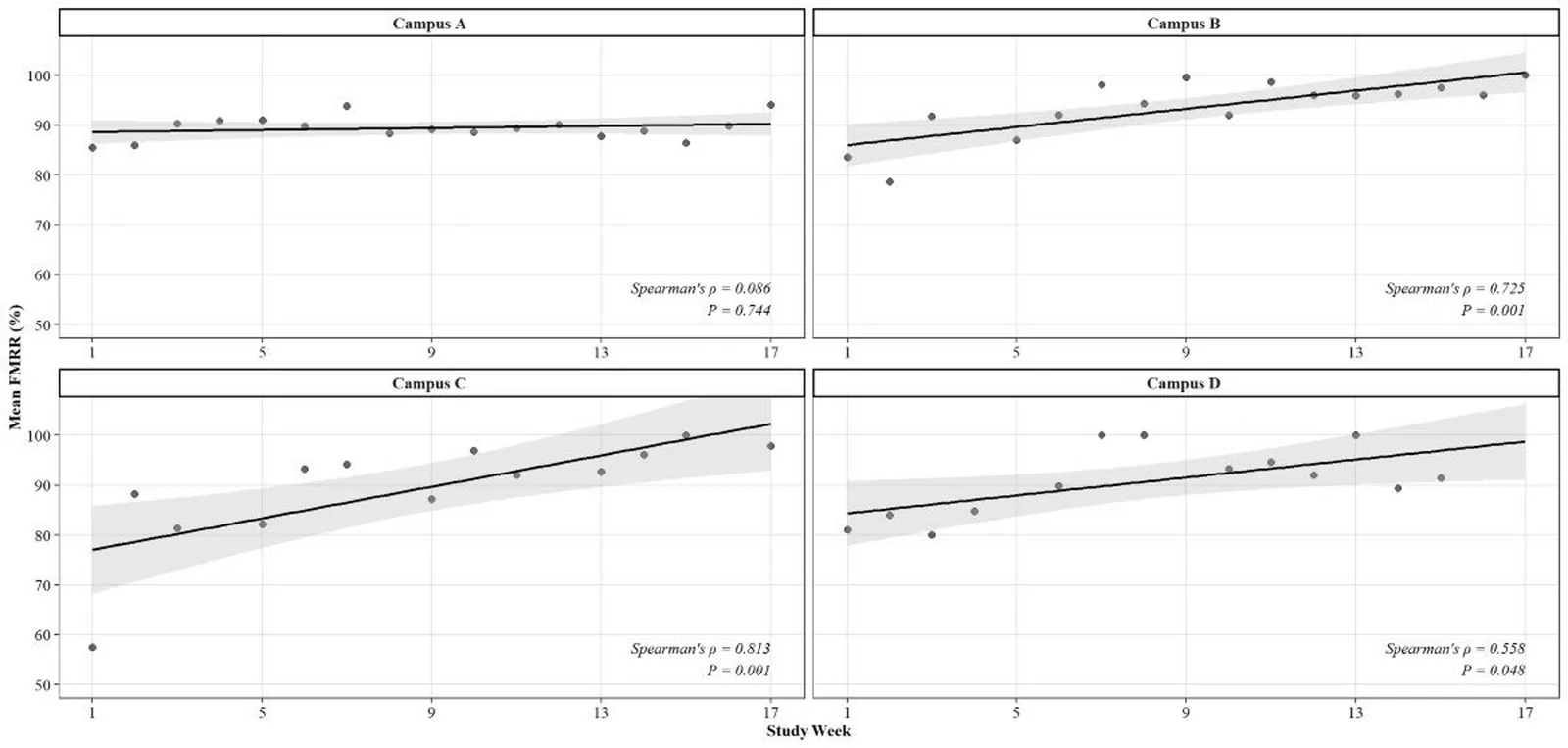
Temporal trends of FMRR across four hospital campuses. The scatter plots illustrate the weekly mean FMRR over the 17-week intervention period, stratified by campus (**A**, **B**, **C**, and **D**). Solid circles represent observed weekly mean values. Solid black lines indicate the linear regression fit, while gray shaded regions denote the 95% confidence intervals. Spearman’s rank correlation coefficients (*ρ*) and *P* values are displayed in the bottom-right of each panel to quantify the strength and statistical significance of temporal trends. Campus C showed the strongest positive temporal trend (*ρ* = 0.813, *P* = 0.001), while Campus A maintained relatively stable FMRR levels (*ρ* = 0.086, *P* = 0.744). FMRR: fluorescent marker removal rate

Dynamic trend analysis across campuses suggested reduced between-campus variability during the intervention period. Campus C, which started with lower baseline performance, showed a positive temporal correlation between study week and FMRR (*ρ* = 0.813, *P* = 0.001). Campus A maintained relatively stable FMRR levels without a significant upward trend (*ρ* = 0.086, *P* = 0.744). Campus B also showed an improving trend (*ρ* = 0.725, *P* = 0.001). Campus D showed a positive trend (*ρ* = 0.558, *P* = 0.048), but this finding should be interpreted as exploratory because of the small number of samples from this campus. Collectively, these trends suggest a reduction in between-campus variability, as shown in Fig. 4. In the sensitivity analysis excluding Campus D, the overall temporal improvement remained statistically significant (Spearman’s *ρ* = 0.525, *P* = 0.031), indicating that the main temporal trend was not driven solely by the smallest campus. The significant between-campus differences (Kruskal–Wallis test, *P* < 0.001) and differences across surface materials (Kruskal–Wallis test, *P* = 0.0009) also persisted in the sensitivity analysis.

### Variation across hospital units

**Fig. 5:**
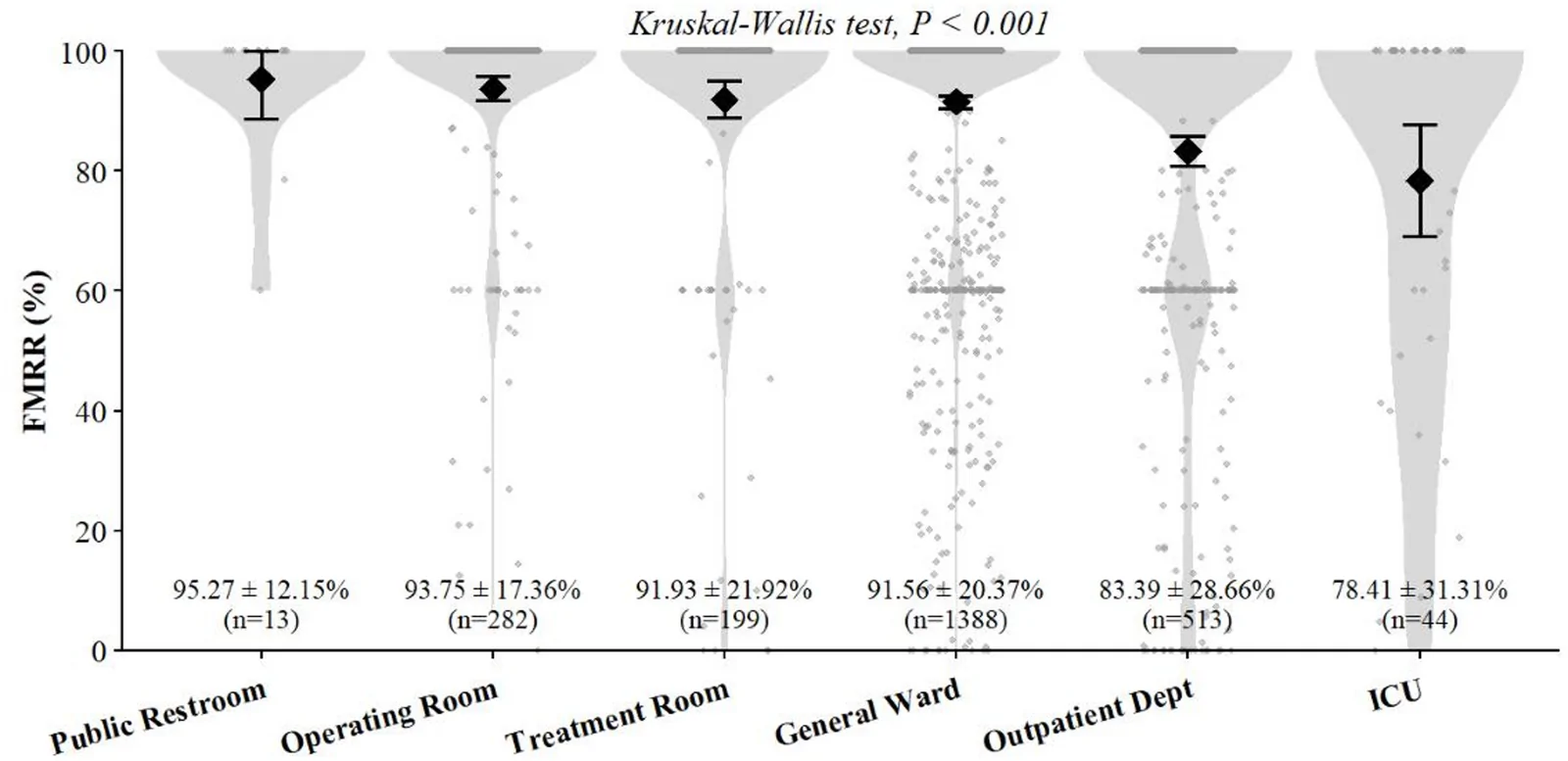
FMRR across different hospital units. Violin plots show the distribution of FMRR in six types of hospital units. The diamond shape (♦) represents the mean, and error bars indicate the 95% CI. Gray jittered points depict individual observations. Numerical labels below each violin display the mean ± standard deviation (SD). Mean ± SD values are shown for descriptive visualization only; inferential comparisons were based on nonparametric tests. A Kruskal–Wallis test indicated significant differences in cleaning performance among units (*P* < 0.001). The concentration of observations at 100% reflects complete marker removal, whereas the concentration around 60% reflects the predefined algorithmic partial-removal tier used when residual fluorescence was present but could not be reliably segmented. FMRR: fluorescent marker removal rate, ICU: intensive care unit, CI: confidence interval

Significant differences in FMRR were observed across clinical and public areas (Kruskal–Wallis test, *P* < 0.001). As shown in Fig. 5, cleaning performance differed across hospital units. Public restrooms and operating rooms showed higher FMRR scores, whereas intensive care units and outpatient departments showed lower and more variable scores, identifying them as priority zones for cleaning improvement. The concentration of observations at 100% reflects complete marker removal, whereas the concentration around 60% reflects the predefined algorithmic partial-removal tier described in Sect.  2.2. Therefore, the unit-level distributions should be interpreted together with the algorithm description and Additional file 1.

Post hoc Dunn’s tests revealed that outpatient departments had significantly lower FMRR than all other clinical units (all *P* < 0.001). Similarly, the FMRR in ICUs was significantly lower than that in operating rooms (*Z* = − 3.94, *P* = 0.001), treatment rooms (*Z* = − 3.63, *P* = 0.004), and general wards (*Z* = − 3.47, *P* = 0.008). Detailed results are provided in Table 2.

**Table 2:** Pairwise comparisons of FMRR between high- and low-performing units

Comparison (Lower vs. Higher Performance)	Z statistic	Adjusted *P* value
Outpatient Departments vs. General Wards	− 6.42	< 0.001
Outpatient Departments vs. Operating Rooms	− 5.93	< 0.001
Outpatient Departments vs. Treatment Rooms	− 4.84	< 0.001
ICUs vs. Operating Rooms	− 3.94	0.001
ICUs vs. Treatment Rooms	− 3.63	0.004
ICUs vs. General Wards	− 3.47	0.008

*P* values were adjusted using the Bonferroni method. The *Z* statistic represents the standardized difference in mean ranks; negative values indicate that the first group in the comparison has a lower FMRR rank than the second group. FMRR: fluorescent marker removal rate; ICUs: intensive care units

### Impact of surface materials

**Fig. 6:**
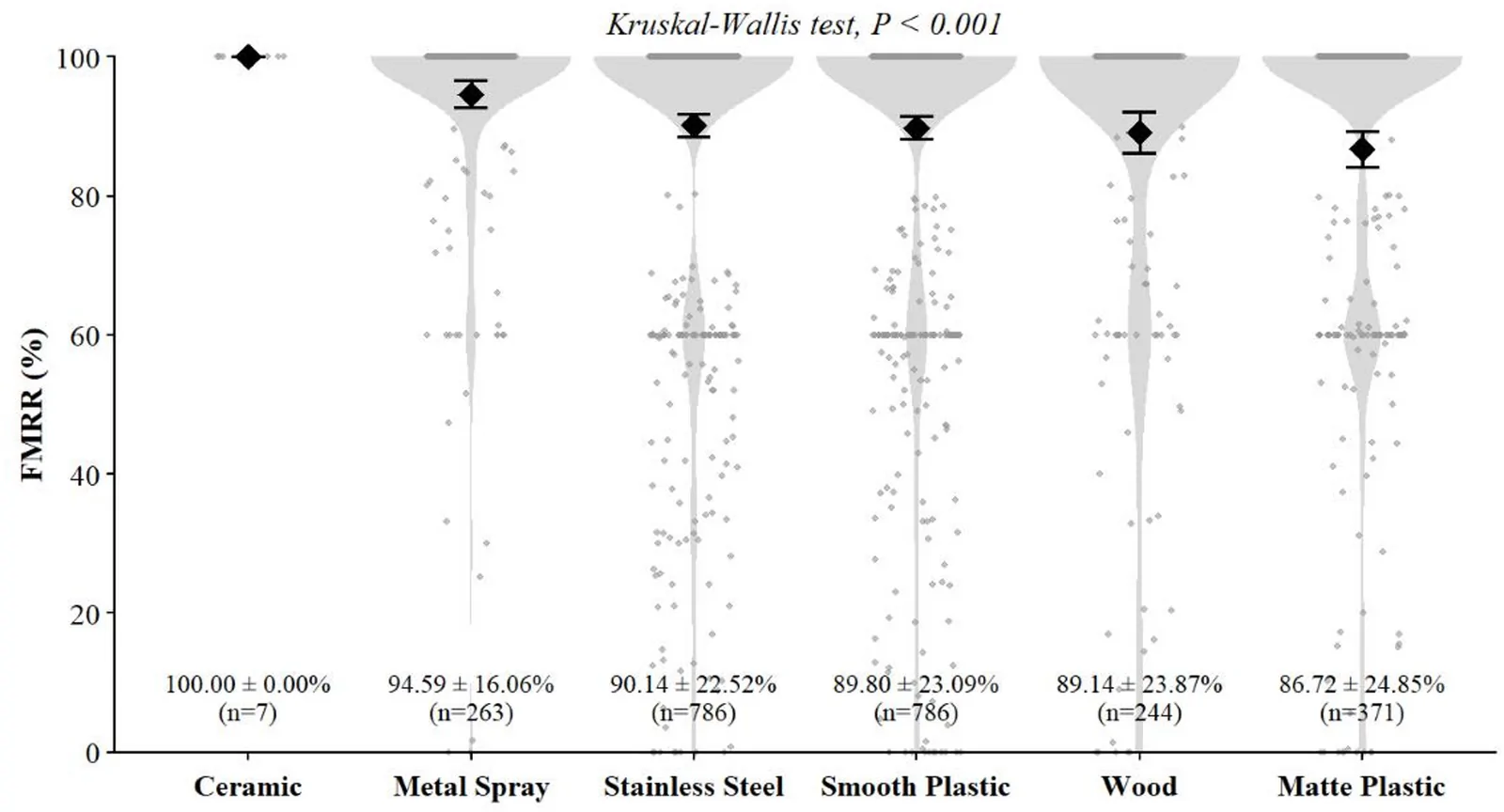
FMRR across different surface material types. Violin plots illustrate the distribution of FMRR for six common hospital surface materials. The diamond shape (♦) represents the mean, and error bars indicate the 95% CI. Gray jittered points depict individual observations. Numerical labels below each violin display the mean ± standard deviation (SD). Mean ± SD values are shown for descriptive visualization only; inferential comparisons were based on nonparametric tests. A Kruskal–Wallis test indicated significant differences in FMRR among materials (*P* < 0.001). The concentration of observations at 100% reflects complete marker removal, whereas the concentration around 60% reflects the predefined algorithmic partial-removal tier used when residual fluorescence was present but could not be reliably segmented (see Methods, Sect.  2.2). FMRR: fluorescent marker removal rate, CI: confidence interval

FMRR varied significantly across different surface materials (Kruskal–Wallis test, *P* < 0.001), suggesting an association between surface material type and cleaning performance. Ceramic surfaces showed the highest FMRR scores in this dataset; however, this finding should be interpreted with particular caution because only seven ceramic samples were included. In contrast, matte plastic and wooden surfaces showed lower and more variable FMRR scores and may represent relatively difficult-to-clean materials (Fig. 6). These findings should be interpreted cautiously because surface material distribution and campus distribution were not fully independent.

Notably, for all materials except ceramic, the violin plots of FMRR displayed a pronounced concentration at 100% alongside a tail of lower scores, reflecting high performance variability. The additional concentration around 60% reflects the predefined algorithmic partial-removal tier described in Sect.  2.2. For smooth surfaces such as stainless steel and glossy plastic, specular reflection may have affected image segmentation or visual recognition of residual fluorescence. For rough or textured surfaces such as matte plastic and wood, surface microstructure may have contributed to incomplete marker removal. These explanations remain hypotheses and require further validation.

## Discussion

### Principal findings and clinical implications

The primary finding of this study is that integrating a computer vision-assisted mobile monitoring tool into the T-PDCA cycle was associated with improved cleaning performance and reduced performance variability across multiple hospital campuses. In addition to improved overall cleaning performance, the weekly mean FMRR remained above 90% after approximately Week 4, although the final-week value should be interpreted cautiously because of the smaller sample size. Campus C showed a strong positive temporal correlation between study week and FMRR (ρ = 0.813), suggesting possible improvement at a previously lower-performing campus. However, campus-specific trends, particularly for smaller campuses, should be interpreted cautiously because of unequal sample sizes.

Persistent environmental contamination has been identified as a key contributor to the nosocomial transmission of multidrug-resistant organisms [16]. In the present study, the FMRR grading thresholds were used as operational quality-improvement thresholds rather than as externally mandated microbiological standards. Specifically, the 80% cutoff was used as a predefined corrective-action threshold, informed by guidance from the Centers for Disease Control and Prevention that uses a thoroughness-of-cleaning rate above 80% as a programmatic benchmark for adjusting ongoing monitoring intensity, as well as by a recent study in which effective cleaning of shared medical equipment was defined as removal of at least 80% of ultraviolet fluorescent dots [14, 15]. The ≥ 90% category was used as a high-reliability performance target rather than as a validated threshold for microbial decontamination or healthcare-associated infection risk reduction. These findings suggest that a structured digital tool embedded within a management framework can support measurable and systematic improvement in environmental cleaning quality, aligning with broader evidence that real-time, data-driven feedback enhances adherence to clinical and operational protocols [17, 18].

### Implications for environmental cleaning monitoring

This study demonstrates that computer vision-assisted fluorescent marker assessment can make environmental cleaning monitoring more standardized and less dependent on visual judgment. Compared with conventional approaches, integrating computer vision-assisted monitoring into the T-PDCA framework demonstrated several methodological advantages.

First, the system may help mitigate the influence of the Hawthorne effect on assessment outcomes. Traditional visual inspection methods, although easy to implement, are highly susceptible to behavioral changes when cleaning staff are aware of being observed, often resulting in artificially inflated performance during supervision [6, 19]. Similar observer effects have been reported in hand hygiene studies, where compliance declines once active observation is withdrawn [20]. By incorporating digital traceability and blinded monitoring, the proposed system enabled the continuous recording of cleaning outcomes even in the absence of direct supervision, thereby promoting more consistent cleaning standards over time.

Second, the approach offered a more robust feedback mechanism. According to the Feedback Intervention Theory proposed by Kluger and DeNisi, the efficacy of feedback depends largely on its timeliness and specificity [21]. Unlike adenosine triphosphate (ATP) bioluminescence assays, which can be costly and subject to considerable variability [22], or time-consuming microbiological culture methods, the present system provides low-cost, immediate, and quantifiable feedback. This improvement is reflected in the enhanced stability of cleaning performance, evidenced by the substantial reduction in the weekly standard deviation of FMRR (from 29.72% to 9.59%).

Finally, replacing human visual interpretation in traditional fluorescent marking with computer vision technology reduced subjectivity in outcome assessment and addressed longstanding challenges related to quantification and inconsistent evaluation criteria [23]. This methodological refinement ensured greater comparability of cleaning performance data across the multi-campus hospital system.

These methodological advantages should be interpreted in the context of a locally developed and operationally implemented monitoring system. Further external validation in other hospital settings is needed to assess the broader applicability and long-term performance of this approach.

### Physical and visual factors influencing cleaning quality

Further analysis of the monitoring data allowed us to identify potential physical and behavioral mechanisms through which surface material characteristics influence cleaning performance.

First, the relatively low FMRR observed on matte plastic and wood surfaces suggests a negative association between surface roughness and cleaning ease. Previous studies have shown that microscopic surface irregularities can provide physical harborages for bacteria and fluorescent markers, enabling them to persist despite routine wiping and resist removal by mechanical shear forces [24, 25]. These findings indicate that standard cleaning procedures may be insufficient for such materials, suggesting that increased physical friction or the replacement of porous materials with smoother alternatives should be considered where feasible.

Second, although smooth surfaces such as stainless steel demonstrated relatively high median FMRR values, their distributions exhibited pronounced left-skewed patterns with long tails of lower scores. We hypothesize that optical interference may contribute to this observation, as highly reflective surfaces can impair visual judgment under ultraviolet illumination, potentially leading to missed areas during cleaning. This finding suggests that training for cleaning smooth surfaces should not focus solely on wiping force or frequency but also incorporate strategies to mitigate visual glare and improve recognition in high-reflectivity environments.

### Area-specific challenges in environmental cleaning

Our analysis further revealed substantial variability in cleaning performance within ICUs and outpatient departments, with standard deviations exceeding 28%, indicating that these settings pose complex challenges for environmental cleaning management. Although ICUs generally adhere to the most stringent cleaning and disinfection standards, they demonstrated the poorest stability in cleaning performance across all areas. This finding is consistent with observations by Munoz-Price et al., who noted that dense equipment configurations and the clinical complexity of critically ill patients can create both physical and cognitive barriers to effective cleaning in ICUs [26].

In contrast, lower cleaning performance in outpatient departments primarily reflects operational constraints in high-throughput environments, particularly in large healthcare facilities with substantial patient volumes. Continuous patient flow markedly reduces the available time window for effective cleaning, thereby compromising the completeness and consistency of cleaning practices. These findings suggest that a uniform, hospital-wide training approach may be insufficient to address the heterogeneous demands of different clinical settings.

Accordingly, more context-specific strategies are required. For ICUs, targeted training programs incorporating scenario-based simulations tailored to complex equipment environments may improve cleaning consistency. In outpatient settings, cleaning protocols should emphasize rapid, segmented completion of cleaning and disinfection tasks for different zones or high-touch items within limited timeframes, rather than relying on rigid, traditional cleaning sequences.

### Practical use in multi-campus cleaning management

Compared with approaches requiring the hospital-wide deployment of fixed sensor networks or the procurement of ATP testing equipment, the smartphone-based application model used in this study may offer practical advantages in terms of implementation feasibility and cost. This approach does not require modification of existing physical infrastructure and can be implemented using mobile devices, thereby lowering initial investment and implementation barriers.

These characteristics may be useful for hospitals with limited resources or for systems undergoing multisite expansion. However, the monitoring tool should be combined with clear feedback and corrective-action mechanisms. In this study, weekly reports, supervisor feedback, and corrective actions for low FMRR results were used to translate monitoring data into practice.

The same feedback structure may also be explored in other hospital support services that rely on visual inspection, such as linen management, medical waste disposal, and sterile supply processing. These potential applications require separate validation.

### Limitations

Several limitations should be acknowledged. First, this was a quasi-experimental quality improvement study without a randomized control group. Although cleaning performance improved during the 17-week intervention, causal inference remains limited, and the long-term sustainability of the effect after active supervision ends requires further follow-up. Second, the study primarily assessed a process measure, the FMRR, rather than microbial contamination or healthcare-associated infection outcomes. Therefore, the observed improvement in FMRR should not be interpreted as direct evidence of reduced pathogen transmission or infection risk. Third, the sample distribution across campuses was uneven, with Campus A contributing most observations and Campuses C and D contributing relatively small numbers of samples. Although Additional file 3 suggests that material composition alone is unlikely to explain the observed between-campus differences, residual confounding cannot be fully excluded because this was not a randomized comparison and surface material distribution and campus distribution were not fully independent. Specifically, Campus A had the highest proportion of matte plastic surfaces, which were identified as relatively difficult to clean, whereas Campus D had the highest proportion of stainless steel surfaces, which were relatively easier to clean. This pattern was opposite to what would be expected if material composition were the main explanation for the between-campus performance gap. Fourth, several technical and operational limitations of the monitoring method should be considered. Images were acquired using multiple smartphone models from three brands and stored in JPEG format; formal validation across a wider range of devices was not performed, and no direct comparison between RAW and JPEG image formats was conducted. Therefore, the possible influence of JPEG compression on borderline segmentation cases cannot be completely excluded. In addition, the FMRR score included an algorithm-defined 60% partial-removal tier for images in which residual fluorescence was present but could not be reliably segmented. This design improved scoring consistency in ambiguous images but means that FMRR should not be interpreted as a purely continuous pixel-area measurement in all cases. Finally, fluorescent markers were applied either on the day before or on the day of cleaning assessment and were not rechecked immediately before cleaning. Incidental attenuation due to routine contact could not be excluded and may have led to overestimation of cleaning performance for some individual surfaces. This limitation of traditional binary fluorescent marker readout was one of the primary motivations for developing the computer vision-based FMRR scoring approach, which enables more standardized characterization of partial cleaning than conventional visual readout. Future studies should validate the algorithm against independent image annotation, microbial outcomes, and wider-field imaging approaches for larger clinical environments.

## Conclusions

This study suggests that a computer vision-assisted mobile monitoring system embedded in a T-PDCA framework can make targeted high-touch surface monitoring more standardized, quantitative, and feedback-driven. The proposed approach was associated with improved cleaning performance and greater consistency across multiple hospital campuses. These findings support the use of digital monitoring combined with structured feedback to improve environmental cleaning management in multi-campus hospital systems.

## Supplementary Information

Below is the link to the electronic supplementary material.

Supplementary Material 1

Supplementary Material 2

Supplementary Material 3

## Data Availability

The summary data and supporting materials related to this article are included within the article and its additional files. The de-identified underlying dataset used for the analyses is available from the corresponding author on reasonable request.

## Abbreviations

ATP: Adenosine triphosphate
CI: Confidence interval
FMRR: Fluorescent marker removal rate
ICU: Intensive care unit
IPC: Infection prevention and control
IQR: Interquartile range
JPEG: Joint Photographic Experts Group
LED: Light-emitting diode
LOESS: Locally estimated scatterplot smoothing
PDCA: Plan-Do-Check-Act
SD: Standard deviation
SQUIRE: Standards for quality improvement reporting excellence
T-PDCA: Technology-empowered Plan-Do-Check-Act
UVA: Ultraviolet-A
