# Supplementary material for: Improving environmental cleaning quality and consistency in a multi-campus hospital using a computer vision-assisted mobile application: a prospective interventional study
Source: Antimicrob Resist Infect Control. 2026 Aug 10;15:139. doi: 10.1186/s13756-026-01803-x (PMC13746732; doi:10.1186/s13756-026-01803-x)
Supplement: Supplementary file 1 [file 13756_2026_1803_MOESM1_ESM.docx]

**Additional file 1.** Distribution of FMRR samples across the three algorithm evaluation tiers.

| **Evaluation tier** | **Definition** | **n** | **%** |
| --- | --- | --- | --- |
| Tier 1: Thoroughly cleaned | FMRR = 100% (no residual fluorescence detected above segmentation threshold) | 1963 | 79.89 |
| Tier 2: Partial-removal tier | FMRR = 60% (residual visibly present but precise area not reliably quantifiable) | 150 | 6.11 |
| Tier 3: Continuously quantified | FMRR computed as (1 − residual/initial area) × 100% | 344 | 14.00 |
| **Total** |  | **2457** | **100.00** |

*Note: The algorithm applies a tiered evaluation logic. Tier 1 (FMRR = 100%) reflects complete marker removal. Tier 2 (FMRR = 60%) is a predefined partial-removal value assigned when residual fluorescence is present but cannot be reliably quantified. Tier 3 comprises samples for which FMRR was computed continuously from the residual-to-initial fluorescent area ratio. Data include all valid samples analyzed in the main study. FMRR: fluorescent marker removal rate.*
