# Supplementary material for: Improving environmental cleaning quality and consistency in a multi-campus hospital using a computer vision-assisted mobile application: a prospective interventional study
Source: Antimicrob Resist Infect Control. 2026 Aug 10;15:139. doi: 10.1186/s13756-026-01803-x (PMC13746732; doi:10.1186/s13756-026-01803-x)
Supplement: Supplementary file 2 [file 13756_2026_1803_MOESM2_ESM.docx]

**Additional file 2.** Technical protection and system ownership.

The computer vision-assisted environmental cleaning monitoring system evaluated in this study is supported by the following intellectual property registrations owned by the authors' institution.

**Additional figure 1. Invention patent**

A computer vision-based cleanliness detection system and method. Qian Xiang; Yu Lv; Yunhai Zhang; Bo Zhang. China Invention Patent No. ZL202310797218.3, granted on September 26, 2023.

**Additional figure 2. Utility model patent**

A light-shielding device for fluorescent marker detection. Qian Xiang; Yu Lv; Yunhai Zhang; Bo Zhang. China Utility Model Patent No. ZL202321645587.2, granted on August 15, 2023.

**Additional figure 3. Software copyright registration**

Computer software copyright registration certificate: Intelligent Evaluation System for Fluorescent Marker-Based Environmental Cleaning Effect (Version 1.0). Registration No. 2023SR1046105.
