# Supplementary material for: Improving environmental cleaning quality and consistency in a multi-campus hospital using a computer vision-assisted mobile application: a prospective interventional study
Source: Antimicrob Resist Infect Control. 2026 Aug 10;15:139. doi: 10.1186/s13756-026-01803-x (PMC13746732; doi:10.1186/s13756-026-01803-x)
Supplement: Supplementary file 3 [file 13756_2026_1803_MOESM3_ESM.docx]

**Additional file 3.** Cross-tabulation of surface material composition by campus (counts and row percentages).

| **Campus** | **Ceramic** | **Matte Plastic** | **Metal Spray** | **Smooth Plastic** | **Stainless Steel** | **Wood** | **Total** |
| --- | --- | --- | --- | --- | --- | --- | --- |
| **A** | 0 (0.0%) | 310 (16.9%) | 187 (10.2%) | 577 (31.5%) | 587 (32.1%) | 169 (9.2%) | 1830 |
| **B** | 0 (0.0%) | 41 (10.1%) | 61 (15.1%) | 112 (27.7%) | 132 (32.6%) | 59 (14.6%) | 405 |
| **C** | 7 (4.3%) | 14 (8.5%) | 12 (7.3%) | 80 (48.8%) | 41 (25.0%) | 10 (6.1%) | 164 |
| **D** | 0 (0.0%) | 6 (10.3%) | 3 (5.2%) | 17 (29.3%) | 26 (44.8%) | 6 (10.3%) | 58 |
| **Total** | **7** | **371** | **263** | **786** | **786** | **244** | **2457** |

*Note: Cell values are presented as count (row percentage). Row percentages reflect the proportion of each surface material within a given campus. In this dataset, Campus A contained the highest proportion of matte plastic (16.9%), which was associated with lower FMRR scores in the main analysis, whereas Campus D had the highest proportion of stainless steel (44.8%), which was associated with relatively higher FMRR scores. This pattern suggests that material composition alone is unlikely to explain the observed between-campus differences, although residual confounding cannot be fully excluded. Data include all valid samples analyzed in the main study.*
